# *Staphylococcus aureus* bacteremia in alcoholics

**DOI:** 10.1371/journal.pone.0298612

**Published:** 2024-05-21

**Authors:** Klaus Kessel, Erik Forsblom, Eeva Ruotsalainen, Asko Järvinen

**Affiliations:** Division of Infectious Diseases, Inflammation Center, Helsinki University Hospital, Helsinki, Finland; Kwame Nkrumah University of Science and Technology Faculty of Pharmacy and Pharmaceutical Sciences, GHANA

## Abstract

**Background:**

Alcoholism associates with increased *Staphylococcus aureus* bacteremia incidence and mortality. The objective was to compare disease progression, treatment and prognosis of *Staphylococcus aureus* bacteremia in alcoholics versus non-alcoholics.

**Methods:**

The study design was a multicenter retrospective analysis of methicillin-sensitive *Staphylococcus aureus* bacteremia with 90-day follow-up. Patients were stratified as alcoholics or non-alcoholics based on electronic health record data. Altogether 617 *Staphylococcus aureus* bacteremia patients were included of which 83 (13%) were alcoholics.

**Results:**

Alcoholics, versus non-alcoholics, were younger, typically male and more commonly had community-acquired *Staphylococcus aureus* bacteremia. No differences in McCabe´s classification of underlying conditions was observed. Higher illness severity at blood culture sampling, including severe sepsis (25% vs. 7%) and intensive care unit admission (39% vs. 17%), was seen in alcoholics versus non-alcoholics. Clinical management, including infectious disease specialist (IDS) consultations and radiology, were provided equally. Alcoholics, versus non-alcoholics, had more pneumonia (49% vs. 35%) and fewer cases of endocarditis (7% vs. 16%). Mortality in alcoholics versus non-alcoholics was significantly higher at 14, 28 and 90 days (14% vs. 7%, 24% vs. 11% and 31% vs. 17%), respectively. Considering all prognostic parameters, male sex (OR 0.19, p = 0.021) and formal IDS consultation (OR 0.19, p = 0.029) were independent predictors of reduced mortality, whereas ultimately or rapidly fatal comorbidity in McCabe´s classification (OR 12.34, p < 0.001) was an independent predictor of mortality in alcoholics.

**Conclusions:**

Alcoholism deteriorates *Staphylococcus aureus* bacteremia prognosis, and our results suggests that this is predominantly through illness severity at bacteremia onset. Three quarters of *Staphylococcus aureus* bacteremia patients we studied had identified deep infection foci, and of them alcoholics had significantly less endocarditis but nearly half of them had pneumonia.

## Introduction

Alcohol use disorders and alcoholism affects 283 million people worldwide [1]. Alcoholism increases the occurrence of various infectious diseases such as bacterial pneumonia and blood culture-positive infections. Furthermore, alcoholism deteriorates prognosis and increases mortality in bacterial pneumonia and blood culture-positive infections [2–5]. The connection between alcoholism and increased risk and severity of infections is presented as multifactorial [2, 6]. Previous studies suggests that alcohol impairs the immune system through cellular and molecular modifications, including macrophage and neutrophil disturbance, lung surfactant and ciliary dysfunction [6, 7]. Studies associate alcohol abuse with malnutrition, poor dental hygiene and increased aspiration risk through blunted cough reflexes–factors lowering the threshold for infections [5, 6, 8]. Alcoholism has been hypothesized to create a state of subclinical immunocompromise, increasing the risk for severe invasive infections [8].

*Staphylococcus aureus* causes severe bacteremia (SAB) and is associated with significant morbidity and mortality [9, 10]. The incidence of SAB is high in alcoholics [4] and alcoholism is a common comorbidity among SAB patients, with reports of up to 14% of SAB patients suffering from alcoholism [11–14]. Several reports demonstrate that alcoholism is an independent predictor of mortality in SAB [11, 15]. Disease progression and prognosis in SAB are impacted by age and comorbidities [16], the severity of illness [17], deep infection foci, e.g., endocarditis [18, 19]. Appropriate clinical management and infectious disease specialist (IDS) consultation [20] may improve outcomes. Formal IDS consultation has been suggested as a mandatory part of SAB management [21, 22]. However, despite knowledge that alcoholism is a risk factor for acquiring SAB, alcoholism is common among SAB patients and alcoholism deteriorates prognosis in SAB, there is no data on how the disease among alcoholics differs from non-alcoholics.

The objective of our study was to explore the disease progression, treatment and prognosis of methicillin-sensitive (MS) SAB in alcoholics compared to non-alcoholics and to evaluate whether alcoholism would serve as a marker for risk stratification of *Staphylococcus aureus* bacteremia patients. The study was conducted in a low-resistance area and the inclusion of only MS-SAB enabled each patient to receive appropriate antimicrobial therapy without differences in empirical antibiotic choice.

## Materials and methods

### Study population

This was a multicenter retrospective cohort study with 90 days of follow-up, including all adult patients with at least one blood culture positive for MS-SAB from five university and seven central hospitals in Finland during January–May 1999 and January 2000 –August 2002. Furthermore, as an extension, each adult patient with positive blood culture for MS-SAB from Helsinki University Hospital in Finland from January 2006 –December 2007 was included. Patient data was retrieved from both written (years 1999–2002) and electronic (years 2006–2007) hospital archives. We decided to include two time periods to exclude any unknown temporary differences in personnel arrangements or treatment practices. Any possible disadvantage with either information storage pattern was accounted for by including both paper and electronic hospital records. Patients and corresponding *Staphylococcus aureus* bacterial isolates were matched through the unique personal number provided to all Finnish residents. A total of five cases of methicillin-resistant SAB were excluded. Data collection included gender, age, bacteremia acquisition, underlying diseases, length and administration route of any antibiotic therapy. Any pre-bacteremia abuse of alcohol was documented. Infection focus documentation was based on either clinical suspicion only or verified by radiological, bacteriological, or pathological investigations. Laboratory results and time to defervescence (axillary temperature < 37.5°C) and hospital and ICU (intensive care unit) duration were recorded.

#### Ethics statement

The institutional review board of Helsinki University Hospital, The Ethical committee of Helsinki University Central Hospital and each study site approved the study (study number 82/99). This was a retrospective study, hence, no written or verbal consent was required.

### Definitions

Hospital-acquired bacteremia was defined as SAB with the first positive blood culture for *Staphylococcus aureus* obtained (1) ≥ 48 h after hospital admission or (2) within 48 h of hospital admission with a preceding hospital discharge in the past 7 days. We applied McCabe’s criteria to classify the severity of each patient’s underlying diseases as *healthy or nonfatal*, or as *ultimately or rapidly fatal* [23]. The modified Duke criteria were applied to define endocarditis [24]. Sepsis in connection with hypotension, hypoperfusion or organ failure was classified as severe sepsis, and sepsis with arterial hypotension despite adequate fluid resuscitation was defined as septic shock [25]. IDS consultation within 7 days of the first positive blood culture for *Staphylococcus aureus* was documented and categorized into formal bedside consultation, informal telephone consultation or no consultation [26].

### Diagnosis of alcoholism

Alcoholism was either determined by the treating physician or by our research team. In the latter case we looked at patients’ electronic (or written) health record (EHR) and applied the WHO´s clinically practical definition to determine alcoholism, namely “*chronic continual drinking or periodic consumption of alcohol which is characterized by impaired control over drinking*, *frequent episodes of intoxication and preoccupation with alcohol and the use of alcohol despite adverse consequences*” [27]. Due to the retrospective nature of the study, we did not use exact diagnostic entities for alcoholism defined by diagnostic criteria in the ICD-10 [28]. When we used a patient’s EHR (or written records) to determine alcoholism we looked at (1) records of previous visits to outpatient alcohol rehab clinics, (2) repeated visits to the emergency room due to alcohol-related issues, or (3) past medical history taken by the treating physician indicating alcoholism.

### Antibiotic therapy

The standard antibiotic therapy was the semisynthetic anti-staphylococcal penicillin cloxacillin. First-line therapy in patients with a penicillin allergy or other contradictions to cloxacillin was a cephalosporin (either cefuroxime or ceftriaxone), and second-line therapy for these patients was vancomycin, clindamycin or carbapenems. In some cases, rifampicin (RMP) and/or a fluoroquinolone were provided as adjunctive therapy. The antibiotic therapy duration was regarded as correct when given intravenously for at least 28 days for a deep infection focus or when given for at least 14 days in the absence of any deep infection. Detailed information on antimicrobial indications, dosages and administration routes has been provided previously [29].

### Outcome

The primary endpoint was mortality at 28 and 90 days. Secondary endpoints were the prevalence of deep infection foci, time to defervescence and length of hospitalization.

### Statistical analyses

Categorical variables were compared with Pearson’s *X* ^2^ test and non-categorical variables with the Student’s *t*-*test*. Odds ratios (OR) with 95% confidence intervals (CI) were calculated. Univariate factors with *p* < 0.1 were entered into multivariate logistic regression model analysis for estimation of parameters associated with outcome. No missing data imputation analyses were performed. The multivariate analyses were performed with available data only. Case fatalities were estimated with Kaplan-Meier graphical presentation. Tests were 2-tailed and *p* < 0.05 was considered significant. Analyses were done with *SPSS 25*.*0*.*0 IBM SPSS Version 25*.*0*. *Armonk*, *NY*: *IBM Corp*.

## Results

### Patient demographics, underlying conditions and predisposing factors

Altogether, of the total 617 SAB patients identified 83 (13%) were defined as alcoholics and 534 (87%) as non-alcoholics (Table 1). Alcoholics, compared to non-alcoholics, were slightly younger on average and more frequently male (Table 1). No differences were observed between the two groups in McCabe´s classification of underlying conditions, however, alcoholics had significantly less cardiovascular (16% vs. 31%) and chronic kidney disease (5% vs. 15%). Alcoholics, compared to non-alcoholics had significantly more acute or chronic liver disease (19% vs. 10%) including higher MELD scores, indicating a higher occurrence of severe or end-stage liver disease. However, no difference was seen in Maddrey´s scores (Table 1). There were not any significant differences in the rates of foreign bodies inserted within the past year. There was a non-significant trend towards less surgical procedures (10% vs. 21%) in the past three months in alcoholics compared to non-alcoholics (Table 1).

**Table 1:** Patient demographics, underlying conditions, and predisposing factors in patients with methicillin-sensitive *Staphylococcus aureus* bacteremia.

Parameters	Total	Alcoholics	Non-alcoholics	OR	P-value
	n = 617	n = 83 (13%)	n = 534 (87%)	(95% CI)	
**Demographics**					
Age (years) > 60, n (%)	287 (47)	29 (35)	258 (48)	0.58 (0.36–0.93)	0.023
Age (years), mean ± SD	617 (100)	55.8 ± 12	57.0 ± 18	‐‐‐	0.464
Male, n (%)	384 (62)	64 (77)	320 (60)	2.25 (1.31–3.87)	0.003
**Underlying conditions**					
Healthy or non-fatal, **^A^** n (%)	436 (71)	56 (67)	380 (71)	0.84 (0.51–1.38)	0.492
Ultimately or rapidly fatal, **^A^** n (%)	181 (29)	27 (33)	154 (29)	1.19 (0.73–1.95)	0.492
Cardiovascular disease, n (%)	181 (29)	13 (16)	168 (31)	0.47 (0.24–0.92)	0.025
Heart valve disease, n (%)	66 (11)	2 (2)	64 (12)	0.22 (0.05–0.91)	0.023
Pulmonary disease, n (%)	77 (13)	8 (10)	69 (13)	0.92 (0.42–2.02)	0.812
Diabetes mellitus, n (%)	108 (18)	16 (19)	92 (17)	1.58 (0.83–3.00)	0.164
Diabetic complications, n (%)	70 (11)	7 (8)	63 (12)	0.86 (0.37–2.01)	0.736
Acute or chronic liver disease, n (%)	67 (11)	16 (19)	51 (10)	3.25 (1.66–6.33)	< 0.001
MELD score ^B^ (points), mean ± SD	67 (11)	16.0 ± 7.1	10.3 ± 4.5	‐‐‐	0.015
Maddrey´s score ^C^ (points), mean ± SD	67 (11)	100 ± 94	87 ± 101	‐‐‐	0.450
Chronic kidney disease, n (%)	83 (14)	4 (5)	79 (15)	0.29 (0.10–0.82)	0.013
Dialysis care, n (%)	65 (11)	3 (4)	63 (12)	0.29 (0.09–0.93)	0.027
Any malignancy, n (%)	63 (10)	6 (7)	57 (11)	0.82 (0.33–2.00)	0.655
Hematological malignancy, n (%)	39 (6)	2 (2)	37 (7)	0.33 (0.08–1.40)	0.115
Immunosuppressive treatment, n (%)	58 (9)	3 (4)	55 (10)	0.40 (0.12–1.32)	0.119
HIV infection, n (%)	8 (1)	0	8 (1)	‐‐‐	‐‐‐
**Predisposing factors, n (%)**					
Injection drug use **^D^**	44 (7)	2 (2)	42 (8)	0.35 (0.08–1.50)	0.141
Prior skin disease or wound **^D^**	234 (38)	29 (41)	194 (37)	1.48 (0.80–2.73)	0.208
Surgical procedure **^E^**	119 (19)	8 (10)	111 (21)	0.49 (0.22–1.08)	0.071
Foreign body **^F^**	112 (18)	13 (16)	99 (19)	1.06 (0.54–2.09)	0.862
Central venous catheter	58 (9)	7 (8)	51 (10)	1.11 (0.47–2.60)	0.814
Any vascular foreign body	82 (13)	8 (10)	74 (14)	0.83 (0.37–1.85)	0.653
Orthopedic foreign body	66 (11)	7 (8)	59 (11)	0.94 (0.40–2.18)	0.876
Prosthetic heart valve	18 (3)	1 (1)	17 (3)	0.46 (0.06–3.51)	0.440

Data are number of patients (%), odds ratios (OR) (95% confidence intervals). HIV, human immunodeficiency virus.

**^A^** Classification according to McCabe and Jackson [23].

^B^ MELD score, Model for End-stage Liver Disease [30].

^C^ Maddrey´s score, assessment of probability for alcohol induced hepatitis [31].

**^D^** During the last six months.

**^E^** Within 90 days.

**^F^** Inserted within the last year.

### Severity of illness and infection foci

Illness severity was greater in alcoholics than non-alcoholics, as alcoholics had higher rates of severe sepsis (25% vs. 7%), intensive care unit (ICU) admission (39% vs. 17%), inotropic therapy (14% vs. 4%), artificial ventilation (13% vs. 4%) and overall higher Pitt bacteremia scores (Table 2 and S1 Fig). In contrast, hospital-acquired SAB was significantly more common in non-alcoholics than alcoholics (56% vs. 37%) (Table 2). However, the total deep infection foci rates in alcoholics and non-alcoholics were similar (71% vs. 76%). Regarding subgroups of deep infection foci, alcoholics had significantly more often pneumonia (49% vs. 35%) and less endocarditis (7% vs. 16%) and less foreign body infections (7% vs 16%) than non-alcoholics (Table 2 and S1 Fig).

**Table 2:** The course, severity, and clinical management of MS-SAB.

Parameters	Total	Alcoholics	Non-alcoholics	OR	P-value
	n = 617	n = 83 (13%)	n = 534 (87%)	(95% CI)	
**Course and severity of disease**					
Pitt bacteremia score > 3	65 (11)	24 (29)	41 (8)	4.93 (2.78–8.75)	< 0.001
Pitt bacteremia score > 5	32 (5)	14 (17)	18 (3)	5.84 (2.78–12.28)	< 0.001
Pitt Score, mean ± SD	602 (98)	1.90 ± 2.62	0.63 ± 1.43	‐‐‐	< 0.001
Emergent dialysis	22 (4)	5 (6)	17 (3)	1.95 (0.70–5.43)	0.194
Inotropic therapy	32 (5)	12 (14)	20 (4)	4.34 (2.04–9.27)	< 0.001
Artificial ventilation	31 (5)	11 (13)	20 (4)	3.93 (1.81–8.53)	< 0.001
Severe sepsis	56 (9)	21 (25)	35 (7)	4.83 (2.65–8.82)	< 0.001
ICU	124 (20)	32 (39)	92 (17)	3.01 (1.84–4.95)	< 0.001
Deep infection foci	464 (75)	59 (71)	405 (76)	0.78 (0.47–1.31)	0.350
Pneumonia	228 (37)	41 (49)	187 (35)	1.81 (1.14–2.89)	0.012
Endocarditis	91 (15)	6 (7)	85 (16)	0.41 (0.17–0.98)	0.038
Mediastinitis	27 (4)	0 (0)	27 (5)	‐‐‐	‐‐‐
Meningitis	7 (1)	1 (1)	6 (1)	1.07 (0.13–9.03)	0.948
Septic arthritis	72 (12)	12 (14)	60 (11)	1.34 (0.69–2.60)	0.395
Osteomyelitis	171 (28)	18 (22)	153 (29)	0.69 (0.40–1.20)	0.187
Septic arthritis or osteomyelitis	212 (35)	27 (33)	185 (35)	0.91 (0.55–1.48)	0.698
Foreign body infection	91 (15)	6 (7)	85 (16)	0.41 (0.17–0.98)	0.038
Deep-seated abscesses	238 (39)	28 (34)	210 (39)	0.79 (0.48–1.28)	0.330
Hospital acquired SAB	328 (53)	31 (37)	297 (56)	0.48 (0.30–0.77)	0.002
Days to defervescence, mean ± SD	580 (94)	7 ± 9	6 ± 9	‐‐‐	0.519
Follow-up (days) **^A^**, mean ± SD	616 (∼100)	69 ± 33	80 ± 25	‐‐‐	0.001
Hospitalization duration (days), mean ± SD	586 (95)	35 ± 27	35 ± 31	‐‐‐	0.988
Fever duration < 7 days	452 (73)	54 (65)	398 (75)	0.76 (0.43–1.34)	0.349
Deceased within 7 days	26 (4)	6 (7)	20 (4)	2.00 (0.78–5.14)	0.780
Deceased within 14 days	49 (8)	12 (14)	37 (7)	2.27 (1.13–4.56)	0.018
Deceased within 28 days	80 (13)	20 (24)	60 (11)	2.51 (1.41–4.44)	0.001
Decreased within 90 days	116 (19)	26 (31)	90 (17)	2.25 (1.34–3.77)	0.002
**Clinical management**					
Bedside consultation **^B^**	520 (84)	64 (77)	456 (85)	0.58 (0.33–1.01)	0.054
Telephone consultation **^B^**	62 (10)	13 (16)	49 (9)	1.84 (0.95–3.56)	0.067
No consultation **^B^**	35 (6)	6 (7)	29 (5)	1.38 (0.55–3.42)	0.492
TTE	204 (33)	28 (34)	176 (33)	1.04 (0.64–1.69)	0.889
TEE	69 (11)	5 (1)	64 (12)	0.47 (0.18–1.21)	0.109
Leukocyte map	176 (29)	27 (33)	149 (28)	1.25 (0.76–2.05)	0.385
Full-body CT	244 (40)	35 (42)	209 (39)	1.13 (0.71–1.81)	0.599
Anti-staphylococcal AB at day 7	488 (79)	58 (70)	430 (81)	0.56 (0.34–0.94)	0.027
Cephalosporin at day 7	92 (15)	16 (19)	76 (14)	1.44 (0.79–2.61)	0.230
Other antibiotic at day 7	37 (6)	9 (11)	28 (5)	2.20 (1.00–4.84)	0.046
Correct antibiotic duration (14–28 days)	356 (58)	36 (43)	320 (60)	0.51 (0.32–0.82)	0.005
Correct antibiotic duration (14–28 days)–adjusted **^C^**	327 (53)	32 (56)	295 (66)	0.65 (0.37–1.13)	0.124
Aminoglycoside treatment	102 (17)	9 (11)	93 (17)	0.58 (0.28–1.19)	0.134
Fluoroquinolone treatment	303 (49)	46 (55)	257 (48)	1.34 (0.84–2.13)	0.216
RMP treatment	363 (59)	35 (42)	328 (61)	0.46 (0.29–0.73)	0.001
RMP treatment at least 14 days, n	290 (47)	27 (33)	263 (49)	0.50 (0.30–0.81)	0.005

Data are number of patients (%), odds ratios (OR) (95% confidence intervals). ICU, intensive care unit; TTE, transthoracic echocardiography; TEE, transesophageal echocardiography; CT, computed tomography; SAB, *Staphylococcus aureus* bacteremia; AB, antibiotic; RMP, rifampicin.

**^A^** Follow-up duration in days until death or discharge.

**^B^** Infectious disease specialist (IDS) consultation within seven days.

**^C^** We left out cases deceased within 28 days in order to control for cases of discontinued antibiotics due to death.

### Clinical management

Altogether, 84% (n = 520) of patients had a formal bedside IDS consultation and 10% (n = 62) had an informal telephone IDS consultation, whereas 6% (n = 35) were managed without IDS consultation (Table 2). No significant differences in distribution of consultation were seen when comparing alcoholics with non-alcoholics. Radiological imaging, including transthoracic echocardiography (TTE), transesophageal echocardiography (TEE), full-body computed tomography (CT) and leukocyte indium-111 scintigraphy (leukocyte map) were provided equally to alcoholics and non-alcoholics (Table 2 and S2 Fig).

All patients were treated with an antibiotic effective against the cultured strain of *Staphylococcus aureus* as soon as the strain’s susceptibility data was available. The vast majority received an anti-staphylococcal penicillin (80%). Alcoholics, compared to non-alcoholics, were treated less often with an anti-staphylococcal penicillin (70% vs. 81%) and more often with a broader spectrum (other) antibiotic (11% vs. 5%) (Table 2 and S2 Fig). No differences were observed in adjunctive fluoroquinolone or aminoglycoside use, whereas alcoholics received less rifampicin (RMP) therapy than non-alcoholics (42% vs 61%). Proper duration of antibiotic therapy was observed less often in alcoholics compared to non-alcoholics (Table 2). However, the high case-fatality among alcoholics may have resulted into shorter antibiotic courses on average. Hence, as a secondary analysis, we excluded patients who deceased within 28 days. In this secondary analysis we found no significant difference in proper duration of therapy (Table 2).

#### Laboratory values

No differences between alcoholics and non-alcoholics were observed in inflammatory markers, including leucocyte count and C-reactive protein, taken at initial blood culture collection and on day 3, 7 and 10 (Table 3). D-dimer levels measured on day 1 were similar in alcoholics and non-alcoholics (Table 3). Alcoholics had higher alanine transaminase (ALAT) levels at initial blood culture collection than non-alcoholics (Table 3).

**Table 3:** Laboratory value means in alcoholics and non-alcoholics with MS-SAB.

Parameters	Alcoholics	Non-alcoholics	P-value
Leuk count at initial blood culture (SD), E9/l	12.6 (8.6)	12.9 (5.9)	0.718
Leuk count day 3 (SD), E9/l	10.8 (6.7)	10.2 (5.0)	0.450
Leuk count day 7 (SD), E9/l	10.0 (4.6)	9.6 (4.2)	0.510
Leuk count day 10 (SD), E9/l	9.4 (4.3)	9.2 (4.1)	0.788
CRP at blood culture (SD), mg/l	180 (114)	174 (109)	0.733
CRP day 3 (SD), mg/l	123 (93)	135 (80)	0.330
CRP day 7 (SD), mg/l	81 (61)	71 (58)	0.328
CRP day 10 (SD), mg/l	79 (57)	59 (54)	0.183
D-dimer day 1 (SD), mg/l	4.7	2.1 (1.5)	0.096
D-dimer day 7 (SD), mg/l	5.3	2.0 (1.7)	0.059
ALAT at blood culture (SD), U/l	322 (1421)	49 (79)	0.015

CRP, C-reactive protein; SD, standard deviation; Leuk, leukocyte; ALAT, alanine aminotransferase.

### Outcome, defervescence and hospitalization

The overall case fatality at 14, 28 and 90 days was 8%, 13% and 19%, respectively. Alcoholics, compared to non-alcoholics, had significantly higher case fatality at 14 days (14% vs. 7%), 28 days (24% vs. 11%) and 90 days (31% vs. 17%) (Table 2 and S3 Fig). However, no differences in hospital duration or defervescence were observed between the groups (Table 2).

Factors associated with 90-day mortality were evaluated separately for alcoholics and non-alcoholics (Tables 4 and 5). In univariate analysis among alcoholics McCabe´s ultimately or rapidly fatal classification (OR 8.88, P < 0.001), Pitt bacteremia scores > 5 (OR 3.63, P = 0.027), endocarditis (OR 13.33, P = 0.004) and lack of IDS consultation (OR 13.10, P = 0.005) were associated with poor prognosis (Table 4). Multivariate analysis associated ultimately or rapidly fatal underlying diseases (OR 12.34, P < 0.001) with poor prognosis, whereas male sex (OR 0.19, P = 0.021) and bedside IDS consultation (OR 0.19, P = 0.029) were associated with better prognosis (Table 4).

**Table 4:** Prognostic factors in alcoholics with MS-SAB according to 90-day mortality.

Parameters	Univariate analysis		Multivariate analysis	
	OR (95% CI)	P-value	OR (95% CI)	P-value
Age (years) > 60	2.56 (0.98–6.70)	0.052	3.62 (0.99–13.15)	0.051
Sex, male	0.40 (0.14–1.16)	0.086	0.19 (0.05–0.78)	0.021
Hospital acquired	0.66 (0.25–1.76)	0.403	‐‐‐	‐‐‐
Pitt Bacteremia Score > 3	2.37 (0.88–6.42)	0.086	1.63 (0.45–5.91)	0.460
Pitt Bacteremia Score > 5	3.63 (1.11–11.91)	0.027	‐‐‐	‐‐‐
Severe sepsis	1.50 (0.53–4.25)	0.439	‐‐‐	‐‐‐
ICU	1.59 (0.62–4.07)	0.337	‐‐‐	‐‐‐
Emergent dialysis	3.59 (0.56–22.91)	0.154	‐‐‐	‐‐‐
Inotropic therapy	1.70 (0.48–5.97)	0.404	‐‐‐	‐‐‐
Artificial ventilation	3.12 (0.86–11.38)	0.075	‐‐‐	‐‐‐
Bedside consultation	0.30 (0.10–0.87)	0.023	0.19 (0.05–0.84)	0.029
Telephone consultation	1.46 (0.43–4.98)	0.546	‐‐‐	‐‐‐
No consultation	13.10 (1.44–118.79)	0.005	‐‐‐	‐‐‐
All deep infection foci	2.10 (0.69–6.44)	0.189	‐‐‐	‐‐‐
Pneumonia	1.30 (0.51–3.29)	0.584	‐‐‐	‐‐‐
Endocarditis	13.33 (1.47–120.92)	0.004	‐‐‐	‐‐‐
Rifampicin treatment	0.80 (0.31–2.06)	0.644	‐‐‐	‐‐‐
Healthy or non-fatal **^A^**	0.11 (0.04–0.32)	< 0.001	‐‐‐	‐‐‐
Ultimately or rapidly fatal **^A^**	8.88 (3.08–25.57)	< 0.001	12.34 (3.28–46.42)	< 0.001

ICU, intensive care unit.

**^A^** Classification according to McCabe and Jackson [23].

**Table 5:** Prognostic factors in non-alcoholics with MS-SAB according to 90-day mortality.

Parameters	Univariate analysis		Multivariate analysis	
	OR (95% CI)	P-value	OR (95% CI)	P-value
Age (years) > 60	3.61 (2.18–5.97)	< 0.001	3.26 (1.81–5.91)	< 0.001
Sex, male	0.90 (0.57–1.42)	0.648	‐‐‐	‐‐‐
Hospital acquired	1.97 (1.21–3.19)	0.005	1.39 (0.75–2.59)	0.298
Pitt Bacteremia Score > 3	4.15 (2.12–8.12)	< 0.001	2.45 (0.78–7.65)	0.124
Pitt Bacteremia Score > 5	5.45 (2.10–14.16)	< 0.001	‐‐‐	‐‐‐
Severe sepsis	4.84 (2.38–9.83)	< 0.001	3.00 (1.01–8.92)	0.048
ICU	2.33 (1.38–3.94)	0.001	1.11 (0.50–2.47)	0.801
Emergent dialysis	2.81 (1.01–7.81)	0.039	‐‐‐	‐‐‐
Inotropic therapy	2.19 (0.82–5.87)	0.109	‐‐‐	‐‐‐
Artificial ventilation	3.51 (1.39–8.86)	0.005	‐‐‐	‐‐‐
Bedside	0.38 (0.22–0.66)	< 0.001	0.22 (0.08–0.61)	0.004
Telephone	1.91 (0.97–3.77)	0.058	0.69 (0.21–2.30)	0.544
No consultation	3.30 (1.50–7.24)	0.002	‐‐‐	‐‐‐
All deep infection foci	1.33 (0.76–2.33)	0.312	‐‐‐	‐‐‐
Pneumonia	3.31 (2.08–5.28)	< 0.001	3.56 (2.02–6.26)	< 0.001
Endocarditis	1.81 (1.04–3.15)	0.035	1.99 (0.95–4.13)	0.067
Rifampicin treatment	0.75 (0.47–1.18)	0.210	‐‐‐	‐‐‐
Healthy or non-fatal **^A^**	0.16 (0.10–0.26)	< 0.001	‐‐‐	‐‐‐
Ultimately or rapidly fatal **^A^**	6.18 (3.81–10.03)	< 0.001	5.169 (2.842–9.403)	< 0.001

ICU, intensive care unit.

**^A^** Classification according to McCabe and Jackson [23].

For non-alcoholics univariate analysis associated age over 60 years (OR 3.61, P < 0.001), McCabe´s ultimately or rapidly fatal classification (OR 6.18, P < 0.001), hospital acquired SAB (OR 1.97, P = 0.005), Pitt bacteremia score > 3 and > 5 (OR 4.15 and OR 5.45, P < 0.001, respectively), ICU treatment (OR 2.33, P = 0.001), pneumonia (OR 3.31, P < 0.001), endocarditis (OR 1.81, P = 0.035) and lack of IDS consultation (OR 3.30, P = 0.002) with poor prognosis (Table 5). Bedside IDS consultation (OR 0.38, P < 0.001) was associated with better prognosis (Table 5). Multivariate analysis associated age > 60 (OR 3.27, P < 0.001), McCabe´s ultimately or rapidly fatal classification (OR 5.17, P < 0.001), severe sepsis (OR 3.00, P = 0.048) and pneumonia (OR 3.56, P < 0.001) with poor prognosis, and bedside IDS consultation (OR 0.22, P = 0.004) with better prognosis (Table 5).

When looking at only SAB patients with acute or chronic liver disease (n = 67), alcoholics compared to non-alcoholics had significantly higher case fatality at 28 days (31% vs 4%) and 90 days (50% vs. 6%). However, when excluding SAB patients with acute or chronic liver diseases, a trend (statistically non-significant) towards higher case fatality was observed in alcoholics (n = 32), compared to non-alcoholics (n = 331), at 28 days (19% vs. 6%) and 90 days (19% vs. 18%).

## Discussion

The main finding of the present study was a higher illness severity at initial blood culture collection in alcoholics versus non-alcoholics. Alcoholics had a case fatality rate during the 90-day follow-up period double that of non-alcoholics. Alcoholics had equally many deep infection foci, but the type of infection foci differed from those of non-alcoholics. Half of alcoholics had pneumonia, but endocarditis was less common among them. Differences with respect to age, demographics, underlying conditions and clinical management between alcoholics and non-alcoholics were marginal. When accounting for all prognostic parameters, alcoholics and non-alcoholics that were treated without bedside infectious disease specialist (IDS) consultation had 5-fold higher odds of a fatal outcome.

The connection between alcoholism and increased risk and severity of infectious diseases, including blood culture positive infections, is well documented [2, 4, 5, 32, 33]. Prior reports have documented that alcoholics make up 7–14% of SAB patients [11–14]. In our material the proportion of SAB patients classified as alcoholics (13%) was in the upper range. We observed a non-significant trend towards more ultimately or rapidly fatal disease classification according to McCabe´s classification in alcoholics, however, when we excluded chronic liver disease this trend shifted to the opposite. Hence, the slightly higher occurrence of McCabe´s ultimately or rapidly fatal disease classification in alcoholics was explained solely by chronic liver disease. This may indicate that alcoholics participate in less outpatient care and thus have more undiagnosed conditions. These observations are in line with reports suggesting that alcoholics, compared to the general population, seek healthcare services less frequently due to psychiatric illness, poorer access and mistrust in healthcare services and lack of a social support network [34, 35].

Previous SAB studies report occurrence of severe sepsis in 14%– 25% and ICU treatment in 10%– 36% of patients [11, 13, 14, 17, 36]. These observations are in line with the results of non-alcoholics in the present study. However, the severity of illness in alcoholics was far greater. One fourth of alcoholics presented with severe sepsis and almost two fifths of alcoholics required ICU treatment. More severe disease already upon presentation is most probably due to delayed health care seeking among alcoholics [34, 35, 37]. Other possible factors that might affect the more severe disease among alcoholics are the effects of chronic alcohol use on immune system and general health. Chronic alcohol abuse is associated with malnutrition, poor dental hygiene and increased aspiration risk, and alcohol induced subclinical immunosuppression [5–8]. Recent studies have connected alcohol abuse and alcohol-related liver diseases to reduced levels of blood cell subgroups including monocytes and natural killer cells, as well as interleukin-1 levels and diminished complement pathway functioning, increasing the risk for infectious diseases [38, 39]. In fact, alcoholics had more pneumonia although deep infection foci were equally common in alcoholics and non-alcoholics.

Considering the whole patient cohort, alcoholics received less narrow spectrum antistaphylococcal antibiotics, less adjunctive rifampicin therapy and more broad-spectrum antibiotic treatment such as clindamycin or carbapenems than non-alcoholics. Furthermore, alcoholics were provided with the correct durations of antimicrobial therapy less frequently than non-alcoholics. The greater use of broad-spectrum antimicrobial therapies in alcoholics is most likely related to the high degree of ICU treatment required in that group. Severely ill patients are more likely to be treated with broad spectrum antimicrobial therapy despite knowledge of the actual pathogen. This “confounding by indication” phenomenon has been described previously [40]. However, when excluding patients who deceased within 7 and/or 28 days, no differences in separate antibiotic regimens or proper duration of therapy were observed between alcoholics and non-alcoholics.

The present study observed an overall case fatality at day 28 and day 90 of 13% and 19%, respectively, which is lower than the mortality figures of 10%– 25% for 30-day mortality and 28%– 39% for 90-day mortality in many previous reports on SAB [11, 13, 17, 19, 21, 36]. However, the case fatality in alcoholics was significantly higher at 30 days (24%) and 90 days (31%) compared to non-alcoholics. These observations are in line with higher mortality from various infectious diseases reported in alcoholics compared to non-alcoholics, including pneumonia, sepsis and bacteremia [2, 5, 41].

There are weaknesses in the present study that must be accounted for when interpreting results.

First, a retrospective study design includes risk for bias due to potential differences in patient groups which we have tried to control for using multivariate analyses. Second, the diagnosis of alcoholism was recorded by the treating physician or determined based on data from patient records, and we did not apply exact diagnostic entities for alcoholism for which there are diagnostic criteria provided in the ICD-10 [28]. This procedure carries the risk for both under and overdiagnosis of potential alcoholics. Third, the patient data was collected in January–May 1999, January 2000 –August 2002 and 2006 –December 2007. The question of whether the clinical patient data in the study is valid to current hospital practice may be raised. Clinical practice and treatment of SAB develops continuously as new research results becomes available, however, the fundamentals of SAB management have remained unchanged throughout the years, including the importance of identification, eradication of deep infection foci and non-delayed optimal antibiotic therapy [16, 42, 43]. The high degree of formal IDS consultation has granted documentation of relevant data, high-standard management and a high proportion of patients with verified deep infection foci. This feature of our data allowed for analysis relevant to current clinical practice, including comparison of the types and rate of deep infection foci between alcoholics and non-alcoholics, something not addressed in previous works [11–13].

## Conclusions

The present study suggests that alcoholics, compared to non-alcoholics, have a higher degree of illness severity at the initial phase of MS-SAB and higher mortality from MS-SAB. Alcoholics, versus non-alcoholics, seem to have more potentially undiagnosed background conditions and delayed hospital arrival which may have contributed to a higher degree of illness severity and higher mortality. Thorough formal IDS consultation-guided clinical management improves MS-SAB prognosis in alcoholics. Alcoholics had an equally high rate of deep infection foci as non-alcoholics, but the type of foci differed between them. Half of alcoholics had pneumonia, but they had fewer cases of endocarditis than non-alcoholics. These findings can help physicians in risk stratification of alcoholic patients with SAB.

## Supporting information

S1 Fig Disease severity in alcoholics and non-alcoholics with MS-SAB. ICU, intensive care unit. *** = P < 0.001; * = P < 0.05.
(TIF)

S2 Fig Clinical management of MS-SAB in alcoholics and non-alcoholics. IDS, infectious disease specialist; TTE, transthoracic echocardiography; TEE, transesophageal echocardiography; CT, computed tomography; Anti-staph, anti-staphylococcal antibiotic; AB, antibiotic; RMP, rifampicin. * = P < 0.05; ** = P < 0.01.
(TIF)

S3 Fig Three-month survival in alcoholics and non-alcoholics with MS-SAB. P = 0.001.
(TIF)

S1 File Dataset. (XLSX)
