# Supplementary figures and images for: Staphylococcus aureus bacteremia in alcoholics
Source: PLoS One. 2024 May 21;19(5):e0298612. doi: 10.1371/journal.pone.0298612 (PMC11108141; doi:10.1371/journal.pone.0298612)

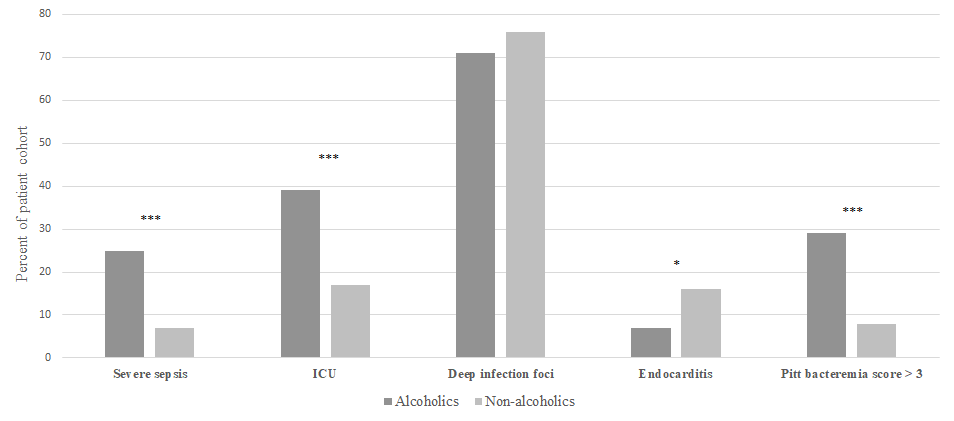

Supplement: S1 Fig — ICU, intensive care unit. *** = P < 0.001; * = P < 0.05. (TIF) [file pone.0298612.s001.tif]

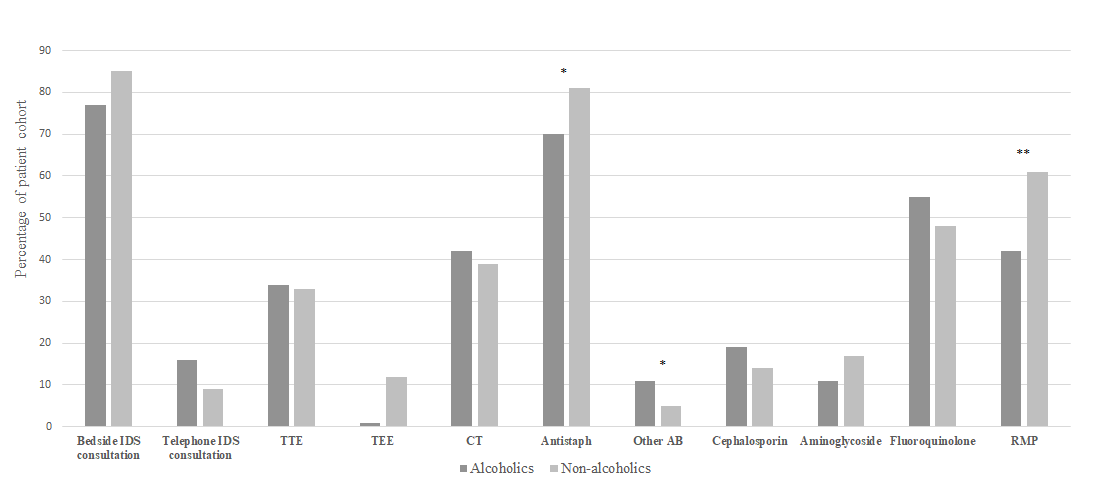

Supplement: S2 Fig — IDS, infectious disease specialist; TTE, transthoracic echocardiography; TEE, transesophageal echocardiography; CT, computed tomography; Anti-staph, anti-staphylococcal antibiotic; AB, antibiotic; RMP, rifampicin. * = P < 0.05; ** = P < 0.01. (TIF) [file pone.0298612.s002.tif]

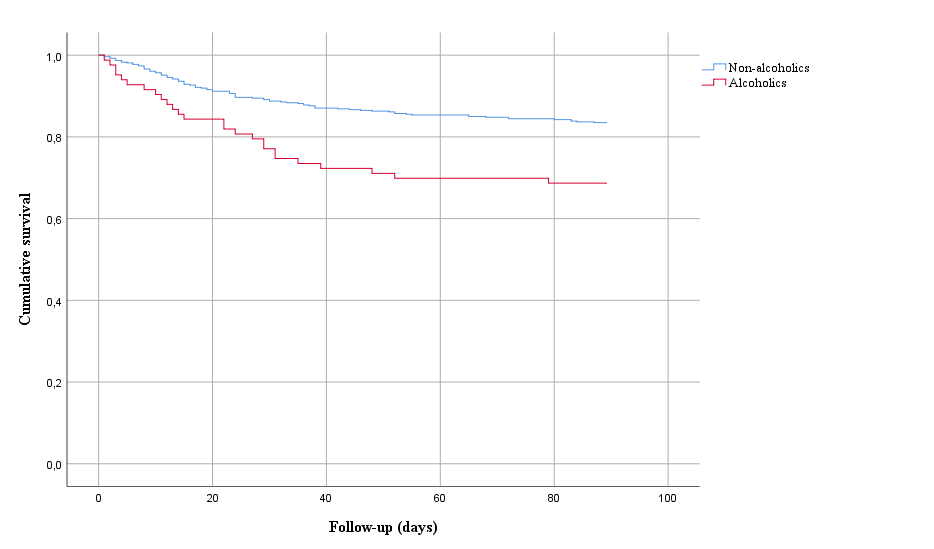

Supplement: S3 Fig — P = 0.001. (TIF) [file pone.0298612.s003.tif]
